# MODIFIED SALTER PELVIC OSTEOTOMY FOR THE DDH TREATMENT

**DOI:** 10.1590/1413-785220233101e259040

**Published:** 2023-04-17

**Authors:** Suvorov Vasyl, Filipchuk Viktor

**Affiliations:** 1 SI -The Institute of Traumatology and Orthopedics by NAMS of Ukraine, Department of Reconstructive Orthopedics and Traumatology in Children and Adolescents, Kyiv, Ukraine.

**Keywords:** Developmental Dysplasia of the Hip, Pelvic Region, Osteotomy, Evaluation of Results of Therapeutic Interventions, Displasia do Desenvolvimento do Quadril, Osteotomia, Pelve, Avaliação de Resultado de Intervenções Terapêuticas

## Abstract

**Objectives:**

Three pelvic osteotomies (Salter, Dega, Pemberton) are widely used in walking patients under seven years old for DDH treatment. We’ve proposed a modified Salter Pelvic Osteotomy (SPO), which has the advantages of the abovementioned osteotomies.

**Methods:**

Short- and mid-term results were assessed in 19 patients after the modified SPO application. Patients were examined before and after the surgery, at 6 months postoperatively, and at follow-up.

**Results:**

Acetabular Index (AI) before the surgery was 39.5 ± 7 °; after the surgery - 24.4 ± 5.5 °, at 6 months - 20.4 ± 5 ° (9-28), at follow-up - 14.5 ± 4 °; AI correction - 14.9 ± 5.5 °. Lateral Centre-Edge Angle at follow-up - 22.7 ± 4.7 °. Clinical results at follow-up were I / II McKay grade in 18 patients (94.7%); radiological results were I / II Severin class in 18 patients (94.7%).

**Conclusion:**

Modified SPO improves the FH coverage in any direction; results after modified SPO are excellent and good in most patients. ***Level of Evidence IV; Case Series***.

## RESUMO

**Objetivos:**

Três osteotomias pélvicas (Salter, Dega, Pemberton) são am-plamente utilizadas em pacientes ambulatoriais com menos de sete anos de idade para tratamento com DDH. Foi proposta a Osteotomia Pélvica de Salter modificada (SPO), que apresenta as vantagens das osteotomias acima mencionadas.

**Métodos:**

Os resultados de curto e médio prazo foram avaliados em 19 pacientes após a aplicação da SPO modificada. Os pacientes foram examinados antes e após a cirurgia, aos 6 meses de pós-operatório, e no acompanhamento.

**Resultados:**

O Índice Acetabular (IA) antes da cirurgia foi de 39,5 ± 7°; após a cirurgia - 24,4± 5,5°, aos 6 meses - 20,4 ± 5° (9-28), no acompanhamento - 14,5 ± 4°; correção da IA - 14,9 ± 5,5°. Ângulo Lateral do Centro-Edge Angle no acompanhamento - 22,7 ± 4,7 °. Os resultados clínicos no acompanhamento foram I / II grau McKay em 18 pacientes (94,7%); os resultados radiológicos foram I / II classe Severin em 18 pacientes (94,7%).

**Conclusão:**

A SPO modificada melhora a cobertura de FH em todos os sentidos; os resultados após a SPO modificada são excelentes e bons na maioria dos pacientes. **Nível de Evidência IV; Série de casos.**

## INTRODUCTION

Developmental Dysplasia of the Hip (DDH) is one of the most common pathologies of the hip joint in children.^1^ The age of DDH detection is critical - non-surgical treatment is effective only in case of early diagnosis (in non-walking patients).^2^ In case of DDH late detection (in walking patients) or after the failure of non-surgical treatment (in case of residual acetabular dysplasia or femoral head redislocation), surgical treatment is indicated.^3^ There are different types of surgeries for DDH management, but the best results were observed after pelvic osteotomies application.^4^

Three different pelvic osteotomies (Salter, Dega, Pemberton) are commonly used in patients with DDH younger than 7 years old.^5^ Each of these osteotomies has certain advantages and disadvantages. Thus, Salter osteotomy is easier to perform, but it is possible to improve only the anterolateral femoral head (FH) coverage and provides lower acetabular deformity correction degree compared to Pemberton and Dega osteotomies.^6,7^ Using Dega osteotomy it is possible to improve the FH coverage in all directions and to achieve a higher correction degree but is technically demanding in patients under 4 years (due to the smaller iliac bone thickness).^7^ Pemberton pelvic osteotomy also allows to achieve higher correction degree, but using it it is possible to improve only the anterolateral FH coverage; another disadvantage after this surgery is a possible triradiate cartilage injury.^6,8,9^

Today it is well-known that DHH presents itself not purely as an anterolateral acetabular deficiency; three types of acetabular deformities were found.^10^ Thus, there is a need for a pelvic osteotomy that would be able to improve FH coverage in all directions. Other prerequisites for pelvic osteotomy are: to ensure a sufficient level of acetabular deformity correction; to be easy to perform regardless of the patient's age; have no risk of triradiate cartilage injury. In our hospital, we use a modified Salter Pelvic Osteotomy (SPO) that meets the abovementioned requirements.

The purposes of this article were:

1. to describe our modification of SPO
2. to show short and middle-term results after this technique

## METHODS

Institutional ethics board committee approval (protocol Nº 4 dated 10.12.2021) was obtained for publishing the results of this investigation.

In our institution modified SPO is used from 2015. It is applied in patients older than 2 years old with acetabular dysplasia (acetabular index(AI) values ≥ 30°); the upper age limit for this technique was 6 years old.

**Figure 1:**
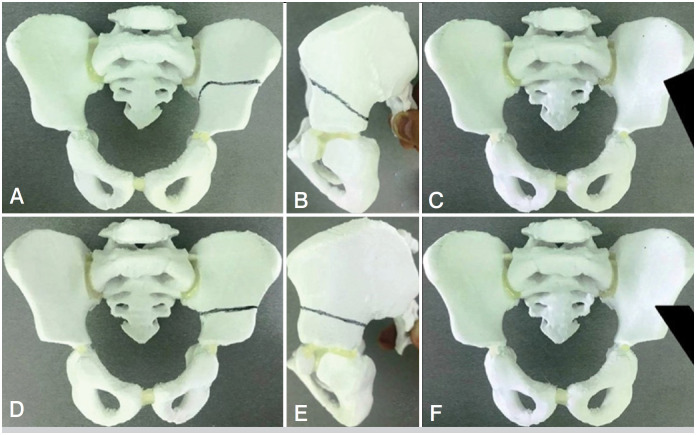
The differences of our SPO modification from the classically described one. In the upper raw (A-C) our modification is shown, in the lower raw (D-F) the classically described SPO is shown.

**Figure 2:**
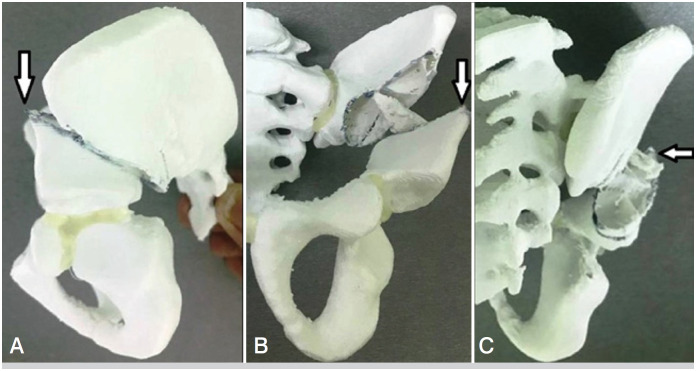
FH coverage improvement in anterior (A), posterior (B) and lateral (C) directions during modified SPO according to applied forces (white arrows).

The differences of our SPO modification from the classically described one^11^ are the following: 1) a curved line of the osteotomy going horizontally up to the terminal line, then it turns downwards (towards the top of the greater sciatic notch) – Figure 1, A/D; 2) more proximal start point of the osteotomy line – Figure 1, B/E; 3) chisel's blade outer side is turned at 45 ° upward laterally (according to the principle of Dega pelvic osteotomy) – Figure 1, C/F. The abovementioned features of our modification are shown in Figure 1. This modified SPO allows to improve the FH coverage in all directions (due to the curved osteotomy line - see Figure 2) and achieve a higher degree of AI correction (due to the turned chisel blade position and, consequently, larger bony contact between iliac bone fragments during acetabular deformity correction). At the same time, our modification is technically easy to perform regardless of the patient's age (since it's itself a complete iliac bone osteotomy and doesn't depend on iliac bone thickness); also, the risk of triradiate cartilage injury is absent (the osteotomy line is far from it). An example of a modified SPO application is shown in Figure 3. To evaluate the results after modified SPO, we’ve selected 19 patients who underwent this surgery for the period 2015-2020.

**Figure 3:**
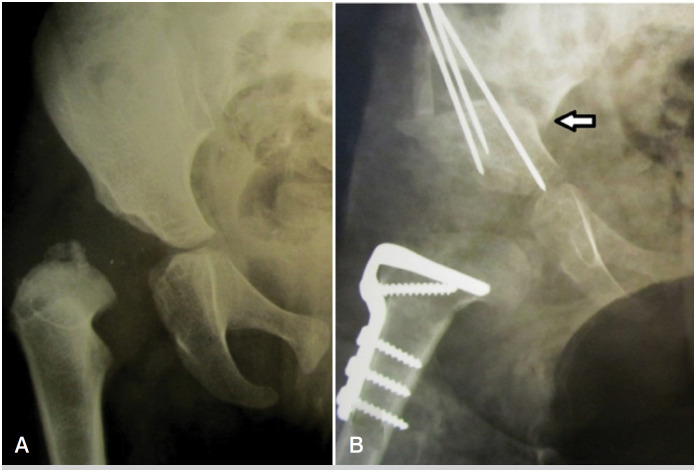
An example of modified SPO application in 3 years old female patient. A – before the surgery, B – after Single Stage Surgery. White arrow points to the upper iliac fragment's sharp angle which is due to curved osteotomy (this is a radiological feature of our modification that is absent in classically described SPO).

Among these patients 18 were girls (94.7%) and 1 was a boy (5.3%); the left hip joint was affected in 12 cases (63.2%), the right - in 7 cases (36.8%); the mean patient's age was 3.6 ± 1.5 years (2-6); the mean follow-up period was 2.7 ± 1.6 years (1-5).

During the pelvic osteotomy, we’ve improved the FH coverage in that direction where it was a deficiency according to X-rays. The deficit of anterior coverage was estimated according to the anterior center-edge angle (CEA) values on the false profile view (in comparison with “healthy” hip). The posterior FH coverage deficit was determined by comparing the anterior and posterior acetabular walls contours on anterior-posterior X-ray (medial position of the posterior wall relative to the anterior was considered as a posterior acetabular wall deficit - as an analog to posterior wall sign in adults). In the case of both anterior and posterior acetabular deficiency, we’ve improved the FH coverage in a more defective direction. Lateral FH coverage was routinely improved in all patients.

Additionally to modified SPO in all cases, we’ve performed femoral varus *derotational osteotomy* (*FVDO*) to decrease femoral anteversion and valgus deformity. Femoral head open reduction was added in case of impossible concentric closed reduction of the FH; femoral shortening was mandatorily performed in these patients. A combination of FH open reduction, pelvic and femoral osteotomies is known as One-Stage Surgery (OSS).^3^ A combination of modified SPO and *FVDO* was performed in 8 patients (42.1%), OSS was performed in 11 patients (57.9%).

Patients were examined clinically and radiologically before, immediately after surgery, at 6 months postoperatively, at followup. Before the surgery, we had determined DDH Tonnis grade and AI values. The day after surgery, we’ve measured AI values and the amount of AI correction. At 6 months postoperatively, we’ve measured AI values and detected any signs (if present) of the FH avascular necrosis (AVN). At follow-up, we’ve determined AI values, Wiberg lateral CEA values; femoral head AVN sequels were assessed according to Bucholz and Ogden.^12^ Long-term radiological results were evaluated according to Severin classification,^3^ long-term clinical results were evaluated according to McKay's criteria.^13^

For statistics calculations we’ve used JASP Team (2020). JASP (Version 0.11.1.0)[Computer software].

## RESULTS

According to DDH Tonnis classification, the II grade was in 6 patients (31.5%), III grade - in 1 patient (5.3%), IV grade - in 12 patients (63.2%).

The AI values before the surgery were 39.5 ± 7 ° (30-53).

The next day after surgery AI values were 24.4 ± 5.5 ° (15-33). The amount of AI correction was 14.9 ± 5.5 ° (8-28).

At 6 months AI values were 20.4 ± 5° (9-28). Signs of femoral head AVN were present in 8 patients (42.1%).

At follow-up AI values were 14.5 ± 4 ° (6-23); lateral CEA values were 22.7 ± 4.7 ° (15-29). Femoral head AVN sequels type I according to Bucholz and Ogden were present in 5 patients (26.3%), type II in 0 patients, type III in 1 patient (5.3%), type IV in 2 patients (10.5%). Clinical results according to McKay's criteria were the following: grade I in 12 patients (63.2%), grade II in 6 patients (31.5%), grade III in 1 patient (5.3%), grade IV in 0 patients (0%). Radiological results according to Severin criteria were the following: class I in 14 patients (73.7%), class II in 4 patients (21%), class III in 1 patient (5.3%), class IV-VI in 0 patients.

Patients’ preoperative characteristics, postoperative clinical and radiological results are presented in Table 1.

**Table 1:** Patients’ preoperative charasteristics, postoperative clinical and radiological results (f-up - follow-up; bef - before; aft - after; diff - difference; 6m - 6 months).

Severin	McKay	AVN	CEA	f-up	6m	diff	aft	bef	AI	Tonnis	f-up	surgery	age	side	sex	Nº
1	1	1	28	6	20	10	22	32		2	5	SPO+FVDO	3	L	F	1
1	1		27	12	16	12	22	34		2	2	SPO+FVDO	6	L	F	2
2	2	1	23	16	20	18	25	43		4	1	OSS	3	L	F	3
1	1		29	11	15	14	15	30		2	2	SPO+FVDO	4	L	F	4
1	1	4	26	12	23	17	26	43		4	3	OSS	6	L	F	5
1	1	4	23	13	26	11	26	37		4	3	OSS	6	R	F	6
3	3		15	20	25	22	31	53		4	1	OSS	2	L	F	7
1	1	1	22	9	9	28	15	43		4	2	OSS	4	L	F	8
1	1		26	18	23	9	27	36		4	2	OSS	6	L	F	9
2	2	1	19	18	23	15	33	48		4	1	OSS	4	R	F	10
1	2		20	16	22	15	27	42		4	1	OSS	3	R	F	11
1	2		16	14	21	8	23	31		4	1	SPO+FVDO	2	R	F	12
1	1	3	27	11	25	14	28	42		4	5	OSS	4	L	F	13
1	1		26	14	12	16	15	31		2	4	SPO+FVDO	2	L	F	14
1	1		27	14	16	11	21	32		2	5	SPO+FVDO	2	R	F	15
1	1	1	24	16	22	13	30	43		3	3	SPO+FVDO	3	L	M	16
2	2		15	23	28	9	31	42		4	4	OSS	2	R	F	17
1	1		24	17	24	26	26	52		4	5	OSS	3	R	F	18
2	2		15	15	18	16	20	36		2	1	SPO+FVDO	3	L	F	19

The long-term result after the modified SPO application is presented in Figure 4.

**Figure 3:**
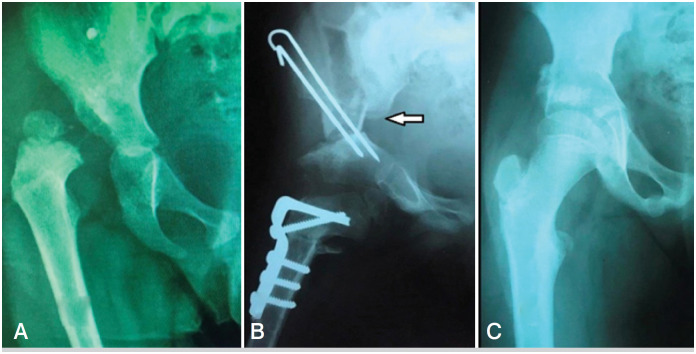
X-rays of 3 years old female patient. A – before the surgery, B – after the Single Stage Surgery (white arrow points to the upper iliac fragment's sharp angle), C – 4 years postoperatively.

Comparison of results after our SPO modification with other authors’ results after standard SPO (or their modifications) is presented in Table 2.

**Table 2:** Comparison of results after modified SPO with other authors’ results.

Author (reference)	Results	
	Radiological	Clinical
Esmaeilnejad- Ganji, S. M. ^1^	Severin І\ІІ - 96.7%; AI - 11.24; CEA - 39.1	Mac-kay І\ІІ - 94.5%
Gurger, M. ^2^	Severin І\ІІ – 100%; AI -18.0; CEA -39.0	Mac-kay І\ІІ – 100%
Bhuyan BK ^3^	Severin І\ІІ - 83.4%; AI - 21°; CEA - 23.5°	Mac-kay І\ІІ - 90%
Ahmed, E. ^13^	Severin І\ІІ – 76.8%; AI - 19°; CEA - 26°	Mac-kay І\ІІ – 80.8%
Da Rocha, V. L. ^14^	Severin І\ІІ - 69.2%, AI – 18.5°	Dutoit ( excellent +good) – 92.3%
Chen Q ^15^	Severin І\ІІ - 83.3%; AI - 21 2°; CEA - 23.5°	Mac-kay І\ІІ – 90%
Xie X ^16^	Severin І\ІІ - 97.6%	Mac-kay І\ІІ - 98.3%
Ahmed K ^17^	Severin І\ІІ - 90%	Mac-kay І\ІІ - 89.5%
Bayhan, I. A. ^18^	Severin І\ІІ – 92%; AI - 12 8°; CEA - 30 9°	Mac-kay І\ІІ - 92%
Morin, C. ^19^	Severin І\ІІ – 96.3%; AI -12 8°; CEA - 29°	Harris hip score - 94.5
Da Rocha, V. L. ^20^	Severin І\ІІ – 88.9%; CEA - 20.7 5.02°	Dutoit (excellent+good) –83.3%
Our modification	Severin І\ІІ – 94.7%; AI – 14.5 4°; CEA–22.7 4.7°	Mac-kay І\ІІ – 94.7%

## DISCUSSION

Non-surgical management of DDH is effective in case of early diagnosis,^2^ but in neglected cases or after non-surgical treatment failure, surgery is mandatory 3. Pelvic osteotomies are proved to be the most effective surgical option for DDH treatment.^4^ Each pelvic osteotomy that is used for DDH treatment in patients younger than 7 years has its strong sides and drawbacks.^5–9^ Also, it is known that in DDH there is not only a deficiency of anterolateral FH coverage, three types of acetabular deformity were described.^10^ Thus, the ideal pelvic osteotomy should improve FH coverage in all directions and should not have known drawbacks. In this paper, we have described our modification of Salter pelvic osteotomy, which meets all the above requirements. Also, the short-term and mid-term results after the modified SPO application were described.

Our modification of SPO differs from the classically described one in that it has a curved line of osteotomy, a more proximal start point of osteotomy and an upward-directed lateral edge of the chisel blade. Short-term and mid-term clinical and radiological results after modified SPO application (follow-up period from 1 to 5 years) were good and excellent in 94.7% of patients; there were no unsatisfactory results. Results after our modification are similar to other authors’ results after the application of standard SPO or their modifications.^1-3,13-20^ However, we believe that it is necessary to individually assess the direction of femoral head deficiency and to consider this during preoperative planning.

Shortcomings of this work are: short follow-up period, absence of control group, patients’ age is limited to 2-6 years old, no results were described after isolated SPO modification (each patient in this study had has additional procedures).

## CONCLUSION

Modified Salter Pelvic Osteotomy make it possible to improve femoral head coverage in any direction in walking patients with DDH under 7 years old; it is technically easy to perform modified SPO independently on the patient's age; this technique provides good Acetabular Index correction; results after modified SPO are excellent and good in the vast majority of patients.
